# The cJUN NH_2_-terminal kinase (JNK) pathway contributes to mouse mammary gland remodeling during involution

**DOI:** 10.1038/s41418-018-0081-z

**Published:** 2018-03-06

**Authors:** Nomeda Girnius, Yvonne J. K. Edwards, Roger J. Davis

**Affiliations:** 1 Program in Molecular Medicine, University of Massachusetts Medical School, Worcester, MA 01605 USA; 2 Howard Hughes Medical Institute, University of Massachusetts Medical School, Worcester, MA 01605 USA

## Abstract

Involution returns the lactating mammary gland to a quiescent state after weaning. The mechanism of involution involves collapse of the mammary epithelial cell compartment. To test whether the cJUN NH_2_-terminal kinase (JNK) signal transduction pathway contributes to involution, we established mice with JNK deficiency in the mammary epithelium. We found that JNK is required for efficient involution. JNK deficiency did not alter the STAT3/5 or SMAD2/3 signaling pathways that have been previously implicated in this process. Nevertheless, JNK promotes the expression of genes that drive involution, including matrix metalloproteases, cathepsins, and BH3-only proteins. These data demonstrate that JNK has a key role in mammary gland involution post lactation.

## Introduction

The mammary gland is dynamically regulated by circulating hormones and paracrine/autocrine cytokines during post-natal development. Estrogen promotes ductal development by epithelial cells in the mammary gland after puberty [1, 2]. In contrast, progesterone and prolactin are critically required for the epithelial development of alveoli and subsequent milk production by the mammary gland in response to pregnancy [3–5]. Weaning causes milk stasis, decreased circulating concentrations of prolactin, and increased expression of cytokines that activate the JAK1/STAT3 signaling pathway, including leukemia inhibitory factor (LIF) [6], interleukin 6 (IL6) [7], and oncostatin M (OSM) [8]. LIF may serve to initiate STAT3 activation that engages an autocrine pathway sustained by STAT3-induced OSM expression [8]. The switch from prolactin-stimulated STAT5 activation to LIF/IL6/OSM-stimulated STAT3 activation drives remodeling (involution) of the mammary gland, including collapse of the epithelial cell compartment and replacement by adipose tissue, to enable return to a quiescent state [9, 10].

The requirement of the LIF/JAK1/STAT3 pathway for mammary gland involution is strongly supported by studies of knockout mice. Deficiency of LIF [6], JAK1 [11], or STAT3 [12] causes a similar delay in mammary gland involution. Targets of STAT3 signaling include pathways of lysosome-mediated cell death involving cathepsins [13] and mitochondrion-mediated apoptotic pathways mediated by members of the BCL2 family [11, 14, 15]. Indeed, it is established that the *Bcl2l11* and *Bmf* genes that encode the pro-apoptotic BH3-only proteins BIM and BMF are direct targets of STAT signaling [11, 15]. Increased *Bcl2l11* and *Bmf* gene expression during involution may result from loss of transcriptional repression by STAT5 and increased transcriptional activity mediated by STAT3 [11, 15]. The importance of *Bcl2l11* and *Bmf* gene induction is confirmed by analysis of knockout mice that show delayed involution [11, 15]. The BH3-only proteins BAD and NOXA are also implicated in involution, but studies of BAD-deficient (*Bad*^*−/−*^) mice and NOXA-deficient (*Pmaip1*^*−/−*^) mice demonstrate that these BH3-only proteins are not essential for involution [15].

Although the LIF/IL6/OSM–JAK1–STAT3 signaling pathway has a key role in involution, this pathway appears to function in collaboration with other signaling pathways that contribute to involution, including TGFβ [16]. Several TGFβ isoforms are expressed at low levels during lactation, but are greatly induced during involution [17]. It is therefore likely that TGFβ signaling during involution may contribute to remodeling of the extracellular matrix during involution and TGFβ may also contribute to mammary epithelial cell death. Indeed, deficiency of TGFβ3 [16] or Smad3 [18] causes decreased cell death during involution, whereas the forced expression of TGFβ3 causes increased cell death during lactation [16].

Other signaling pathways might also contribute to the involution response. For example, loss of survival signaling (e.g., AKT and ERK) caused by cell detachment and loss of signaling by integrins and receptor tyrosine kinases may promote cell death [19–21]. Similarly, increased signaling by stress-activated MAP kinases [22–24] may promote cell death during involution. Indeed, it is established that the stress-activated protein kinases p38 MAP kinase [25] and cJUN NH_2_-terminal kinase (JNK) [26, 27] can promote anoikis of mammary epithelial cells. However, it is not known whether these stress-activated MAP kinases contribute to the involution response.

The purpose of this study was to test whether JNK contributes to mammary gland remodeling during the involution response. JNK is activated during involution [28] and has been mechanistically implicated in the involution response [28]. Interestingly, JNK can promote cell death mediated by BH3-only proteins (BMF and BIM) [29–33] that are known to contribute to cell death during involution [11, 14, 15]. Two JNK isoforms (JNK1 and JNK2) with partially redundant functions are expressed in the mammary epithelium. Developmental studies demonstrate that these JNK isoforms are required for anoikis and the clearance of cells from mammary ducts and terminal end buds [26, 27]. Indeed, deficiency of JNK1 plus JNK2 in the mammary epithelium (but not deficiency of JNK1 or JNK2 alone [34]) causes ductal occlusion by suppressing anoikis [26, 27]. Thus, JNK is required for normal mammary gland development and could contribute to involution.

Previous studies have established that involution defects were not observed in mice lacking JNK1 or JNK2 [34]. We therefore examined mice with compound deficiency of JNK1 plus JNK2 in the mammary epithelium. These JNK-deficient mice exhibited delayed involution. Moreover, JNK deficiency caused major disruption of the gene expression program that mediates involution. Together, these data demonstrate that JNK contributes to the normal mammary gland involution response.

## Results

### JNK is required for efficient mammary gland involution

To study mice with compound disruption of the *Mapk8* gene (encodes JNK1) plus the *Mapk9* gene (encodes JNK2) in the mammary epithelium, we established Control (JNK^WT^) mice (*Wap-Cre*^*−/+*^) and JNK-deficient (JNK^KO^) mice (*Wap-Cre*^*−/+*^ *Mapk8*^*LoxP/LoxP*^ *Mapk9*^*LoxP/LoxP*^). *Wap-Cre* expression is induced in the mammary epithelium during lactation [35] and we found *Cre*-mediated recombination in mammary epithelial cells (Figure S1a). Analysis of genomic DNA demonstrated efficient ablation of the *Mapk8* and *Mapk9* genes in the mammary glands of lactating JNK^KO^ mice (Figure S1b, c).

**Fig. 1:**
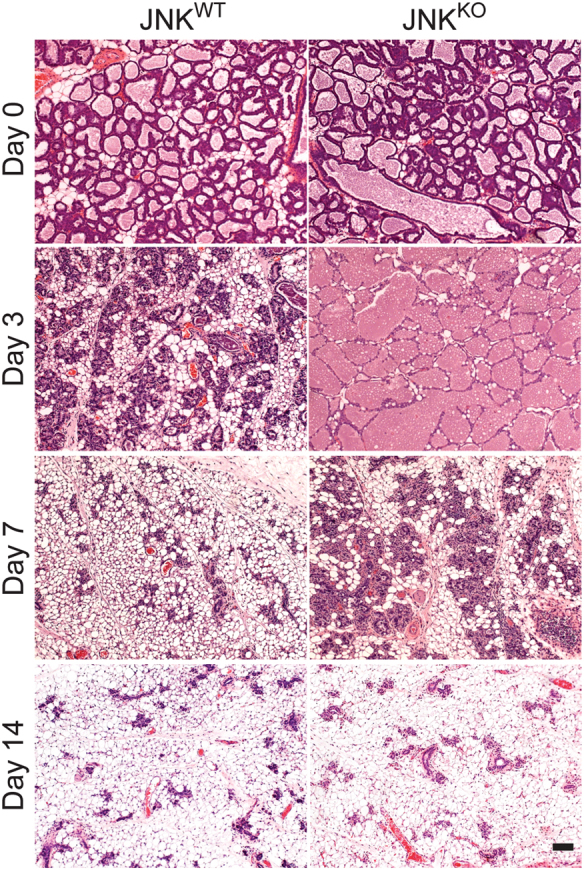
JNK is required for efficient mammary gland involution. Sections of #4 mammary glands from JNK^WT^ and JNK^KO^ mice on involution day 0, 3, 7, and 14 were stained with H&E. The images presented are representative of sections prepared from the mammary glands of 5 JNK^WT^ mice and 5 JNK^KO^ mice for each condition. Scale bar = 100 µm

To test the role of JNK in involution, we examined JNK^WT^ and JNK^KO^ dams that nursed litters for 9 days. Involution was initiated by removal of the pups (involution day 0). Microscopic analysis of tissue sections at this stage demonstrated no differences between the mammary glands of JNK^WT^ and JNK^KO^ mice (Fig. 1; Figure S2). After 3 days of involution, the JNK^WT^ glands exhibited collapse of alveolar structures and the reappearance of adipocytes. In contrast, analysis of the JNK^KO^ glands demonstrated the presence of distended alveoli and no reappearance of adipocytes (Fig. 1; Figure S2). After 7 days of involution, the JNK^WT^ glands morphologically resembled the pre-lactation state, while JNK^KO^ glands still had an expanded epithelial compartment with many collapsed alveoli (Fig. 1; Figure S2). However, on day 14 after the initiation of involution, no differences were detected between the tissue architecture (Fig. 1; Figure S2) or epithelial cell populations (Figure S3) of mammary glands from JNK^WT^ and JNK^KO^ mice. These findings demonstrate that JNK deficiency delays involution, indicating that JNK is required for the proper execution of this process.

### JNK promotes epithelial cell death during involution

**Fig. 2:**
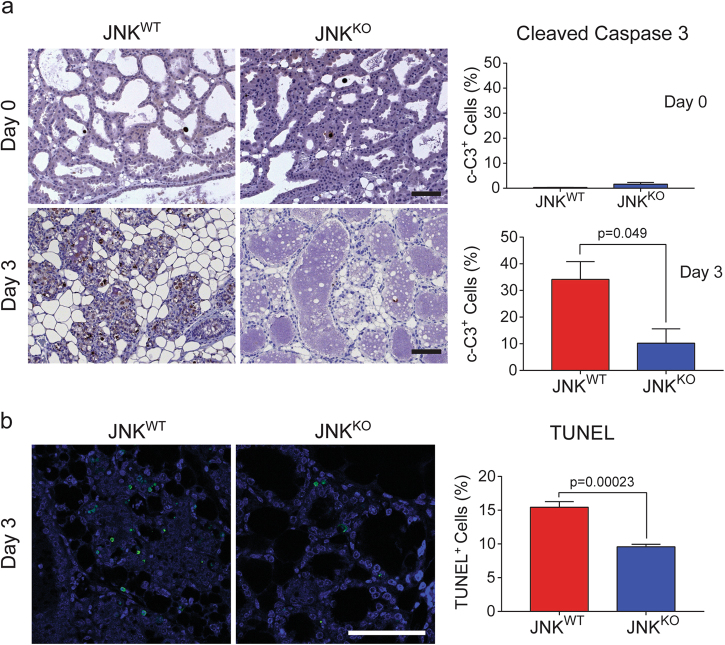
JNK deficiency suppresses cell death during involution. **a** Sections of #4 mammary glands from single parous female mice on involution day 0 or on involution day 3 were stained with an antibody to cleaved caspase 3 (c-C3) and counter-stained with hematoxylin. Representative images are presented. c-C3^+^ cells were quantitated in 6 fields (×40) per section and presented as the % of total cells. Significance was calculated using an unpaired, two-tailed *t*-test (mean ± SEM; day 0, *n* = 5 JNK^WT^ mice and *n* = 4 JNK^KO^ mice; day 3, *n* = 4 JNK^WT^ mice and *n* = 6 JNK^KO^ mice). Scale bar = 100 µm. **b** Sections of #4 mammary glands from mice on involution day 3 were stained by TUNEL assay and counter-stained with DAPI. Representative images are presented. TUNEL^+^ cells were quantitated in 6 fields (×40) per section and presented as the % of total cells. Significance was calculated using an unpaired, two-tailed *t*-test (mean ± SEM; *n* = 4 JNK^WT^ mice and *n* = 5 JNK^KO^ mice). Scale bar = 50 µm

To test whether JNK regulates epithelial cell death in mammary glands during involution, we performed immunohistochemistry using an antibody specific for cleaved caspase 3. On involution day 0, few cleaved caspase 3 positive (c-C3^+^) cells were detected in the mammary glands of JNK^WT^ and JNK^KO^ mice (Fig. 2a). However, on day 3 of involution a substantial increase in the number of c-C3^+^ cells was found in the glands of JNK^WT^ mice, but not JNK^KO^ mice (Fig. 2a). In contrast, large numbers of c-C3^+^ cells were found in both JNK^WT^ and JNK^KO^ mice on day 7 of involution (Figure S4). These data indicate that JNK deficiency delays cell death during involution. To confirm this conclusion, we examined terminal deoxynucleotidyl transferase dUTP nick end labeling (TUNEL) of cells on day 3 of involution. In agreement with the c-C3 data, there were reduced numbers of TUNEL^+^ cells in glands from JNK^KO^ mice compared with JNK^WT^ mice (Fig. 2b). Thus, JNK deficiency causes delayed epithelial cell death in the involuting mammary gland.

### Effect of JNK deficiency on STAT and SMAD transcription factors

**Fig. 3:**
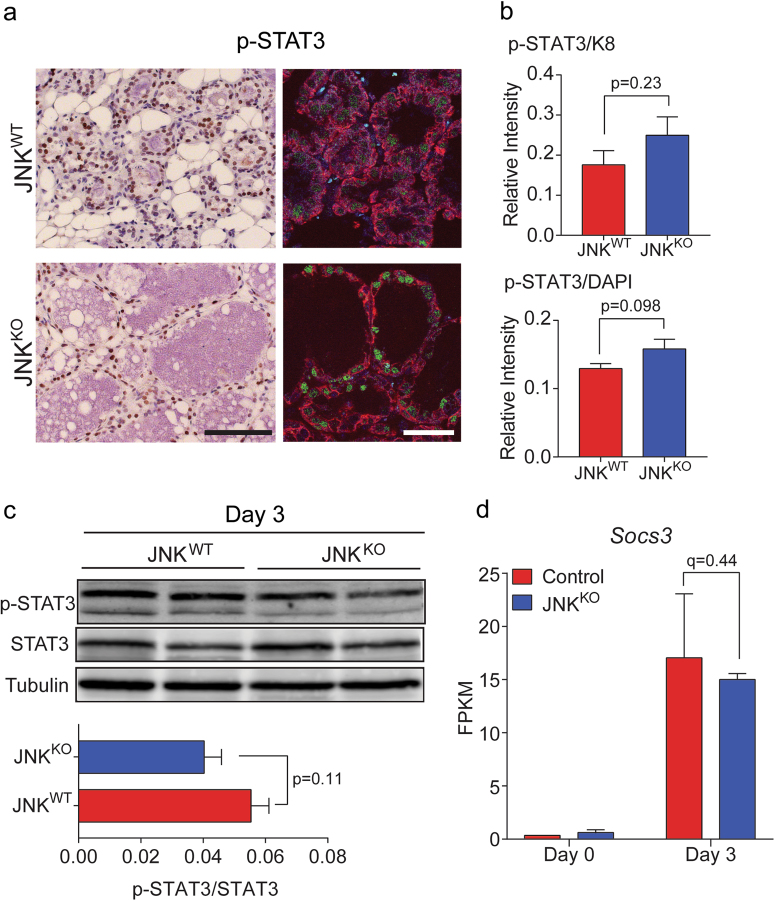
STAT3 signaling is not disrupted in JNK^KO^ glands. **a** Immunohistochemistry (left, scale bar = 100 µm) and immunofluorescence (right, scale bar = 30 µm) were performed on sections prepared from #4 mammary glands of single parous female mice on involution day 3 (*n* = 5 JNK^WT^ mice and *n* = 4 JNK^KO^ mice) using an antibody to phospho-STAT3 (p-STAT3) and counter-stained with hematoxylin or DAPI, respectively. An antibody to keratin 8 (K8) was used to label epithelial cells during immunofluorescence staining. Representative images are presented. **b** K8, p-STAT3, and DAPI fluorescence intensities of involution day 3 glands from JNK^WT^ (*n* = 5 mice) and JNK^KO^ (*n* = 4 mice) were quantitated and p-STAT3 intensity was normalized to K8 and DAPI fluorescence intensity. No significant differences (unpaired, two-tailed *t*-test) between JNK^WT^ and JNK^KO^ glands were detected. **c** Protein extracts prepared from involution day 3 mammary glands were examined by immunoblot analysis by probing with antibodies to p-STAT3, STAT3, and Tubulin. Two representative mice are presented. The quantitative data presented are the mean ± SEM (*n* = 9 JNK^WT^ mice and *n* = 5 JNK^KO^ mice). No significant differences (unpaired, two-tailed *t*-test) between JNK^WT^ and JNK^KO^ glands were detected. **d** The mRNA expression of *Socs3* measured by RNA-seq analysis is presented as the mean FPKM ± SEM; *n* = 3 JNK^WT^ mice and *n* = 3 JNK^KO^ mice per condition. No significant differences between JNK^WT^ and JNK^KO^ glands were detected (calculated by applying the Benjamini–Hochberg method to the *p*-value)

The delayed involution observed in JNK^KO^ mice may be caused by changes in the key transcription factors that regulate this process. It is established that decreased activation of SMAD2/3 or STAT3, or increased STAT5 activation, may cause delayed involution [9, 10]. We therefore examined the activation state of these transcription factors by immunohistochemistry using antibodies to the activating sites of phosphorylation. Studies of phospho-STAT3 (Fig. 3a), phospho-STAT5 (Figure S5a), and phospho-SMAD2/3 (Figure S5b) revealed similar staining of sections prepared from mammary glands of JNK^WT^ and JNK^KO^ mice on day 3 of involution. Indeed, quantitation of phospho-STAT3 immunofluorescence revealed no significant difference between JNK^WT^ and JNK^KO^ glands (Fig. 3b). Immunoblot analysis performed on tissue lysates supported this conclusion (Fig. 3c). Strikingly, the induction of *Socs3*, a STAT3 target gene, was evident after 3 days of involution and was un-affected by JNK deficiency (Fig. 3d). These data demonstrate that the delayed involution caused by JNK deficiency did not reflect disruption of the signaling pathways that regulate the STAT3/5 or SMAD2/3 transcription factors.

### Effect of JNK deficiency on AP1 transcription factors

**Fig. 4:**
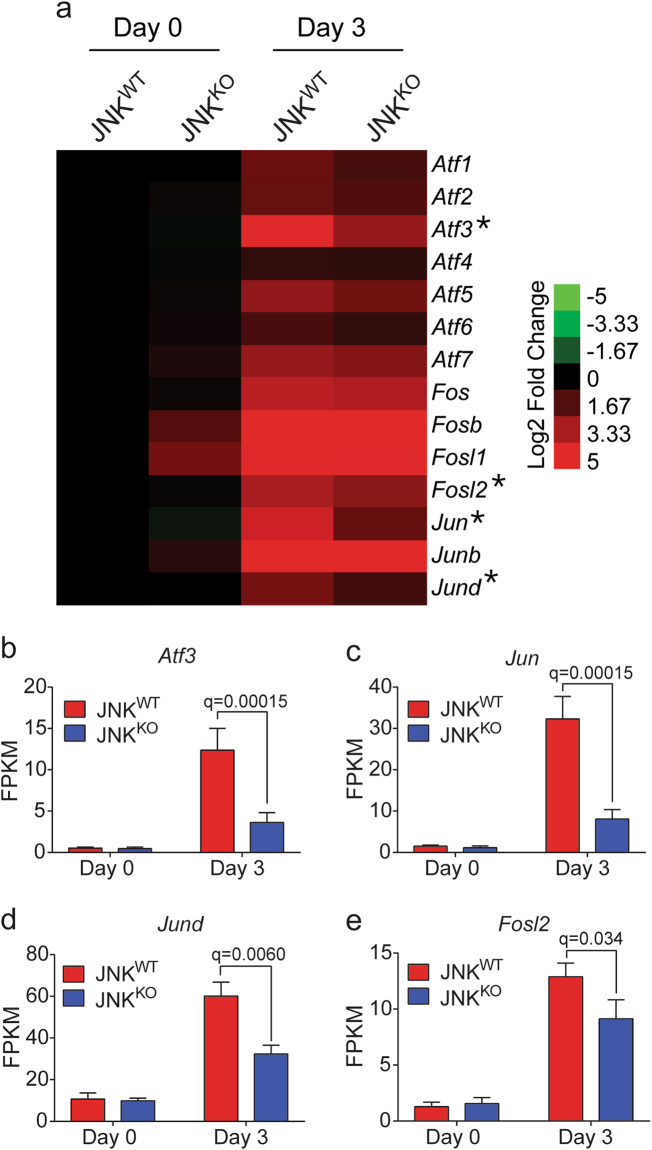
JNK deficiency suppresses the increase in AP1-related transcription factor expression during involution. **a** Heatmap representation of RNA-seq data showing AP1-related transcription factor gene expression. Asterisks denote genes that are differentially expressed (*q* < 0.05) between JNK^WT^ and JNK^KO^ mammary glands on involution day 3 (mean; *n* = 3 JNK^WT^ and *n* = 3 JNK^KO^ mice for each condition). **b**–**e** The mRNA expression of *Atf3* (**b**), *Jun* (**c**), *Jund* (**d**), and *Fosl2* (**e**) measured by RNA-seq analysis is presented as mean FPKM ± SEM; *n* = 3 JNK^WT^ and *n* = 3 JNK^KO^ mice for each condition. The Benjamini–Hochberg method was applied to the *p*-values to calculate *q*-values

Major targets of JNK signaling include members of the Activator Protein 1 (AP1) group of transcription factors that are phosphorylated and activated by JNK [23] and have been implicated in involution [28, 36]. The AP1 family includes members of the JUN and FOS groups, as well as some members of the ATF group of transcription factors. We therefore examined the expression of these AP1-related transcription factors in mammary glands of JNK^WT^ and JNK^KO^ mice on day 0 and day 3 of involution by mRNA sequencing (Fig. 4a). Involution in JNK^WT^ mice caused significantly increased expression of many of these AP1-related transcription factors, including *Atf3*, *Atf5*, *Atf7*, *Fos*, *FosB*, *FosL1*, *FosL2*, *Jun*, *JunB*, and *JunD* (Fig. 4a). Comparison of JNK^WT^ and JNK^KO^ mice demonstrated no significant differences in AP1-related transcription factor expression on day 0 of involution (Fig. 4a). However, on involution day 3 the increased expression of *Atf3*, *FosL2*, *Jun*, and *JunD* detected in JNK^WT^ mice was suppressed in JNK^KO^ mice (Fig. 4b–e). Thus, JNK deficiency causes a selective defect in the AP1-related transcription factor response during involution that is mediated by ATF3, FOSL2, cJUN, and JUND. This observation may be mechanistically relevant to involution because AP1 transcription factor function has been implicated in mammary gland involution [28].

### JNK deficiency disrupts involution-associated gene expression

**Fig. 5:**
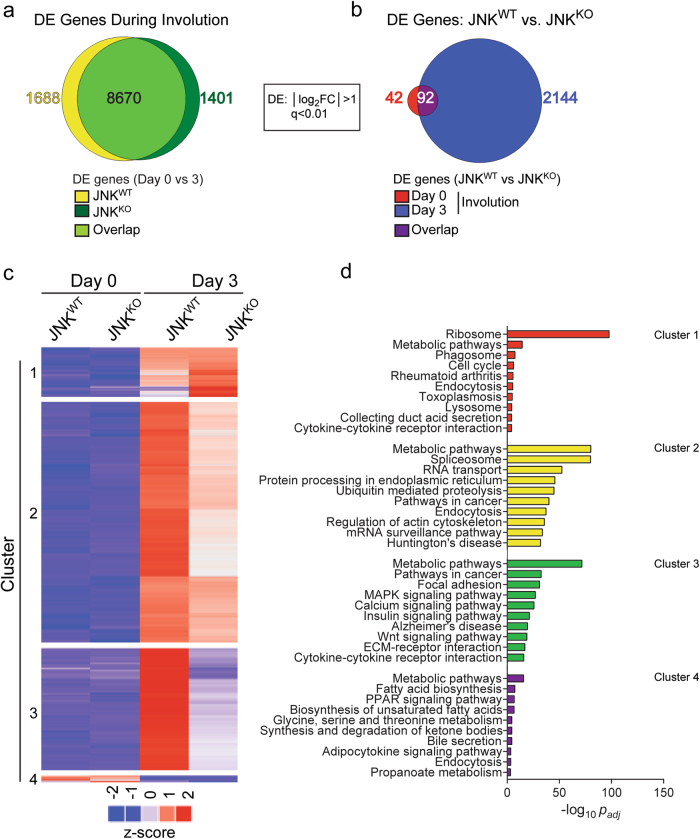
JNK promotes involution-associated gene expression. **a**, **b** RNA-seq analysis of mammary glands of JNK^WT^ and JNK^KO^ mice on involution day 0 or day 3 is presented as a Venn diagram of the number of differentially expressed (DE) genes (|log_2_ Fold Change| > 1; *q* < 0.01; *n* = 3 JNK^WT^ mice and *n* = 3 JNK^KO^ mice for each condition). **c** The heatmap presents *k*-means clustering (*k* = 4) of DE genes in at least one pairwise comparison between JNK^WT^ and JNK^KO^ mammary glands on involution day 0 or day 3. **d** Gene-set enrichment analysis was performed on the four gene clusters. The 10 KEGG pathways identified with lowest *p*_adj_-value (*p*-value adjusted using the Benjamini–Hochberg method) are presented

Comparison of gene expression on day 0 and day 3 of involution demonstrated that 10,358 genes were differentially expressed in JNK^WT^ mammary glands (|log_2_ Fold Change| > 1; *q* < 0.01) (Fig. 5a). A similar number of genes (10,071) were differentially expressed in JNK^KO^ mammary glands (Fig. 5a) and 8620 genes were co-regulated in both JNK^WT^ and JNK^KO^ mammary glands during involution (Fig. 5a). However, 1688 genes were differentially expressed only in JNK^WT^ mice and 1401 genes were differentially expressed only in JNK^KO^ mice. These data demonstrate that involution is associated with major changes in gene expression and that JNK deficiency causes dysregulation of a large fraction (26%) of these genes.

Comparison of RNA expression in mammary glands of JNK^WT^ and JNK^KO^ mice on involution day 0 demonstrated differential expression of 134 genes (|log_2_ Fold Change| > 1; *q* < 0.01) (Fig. 5b), indicating that JNK deficiency causes only small changes in gene expression in the lactating mammary gland. In contrast, comparison of JNK^WT^ and JNK^KO^ mice demonstrated 2236 differentially expressed genes on involution day 3 (Fig. 5b) and only 92 genes were co-regulated during both normal lactation and involution (Fig. 5b). These data indicate that while JNK deficiency causes few changes in gene expression in the lactating mammary gland, JNK deficiency causes major changes in gene expression during involution. The majority of this differential RNA expression (~94%) corresponded to genes that encode proteins (Figure S6).

To characterize involution-associated gene expression in JNK^WT^ and JNK^KO^ mammary glands, we performed *k*-means clustering on the 12,862 genes that were differentially expressed in any of the pairwise comparisons (Fig. 5c). Cluster 1 included genes that were upregulated during involution (day 3) in JNK^WT^ mice and were more strongly upregulated in JNK^KO^ mice. This cluster was highly enriched for ribosomal genes (*p*_adj_ = 1.88 × 10^−98^; Fig. 5d). Clusters 2/3 included genes that were highly upregulated during involution in JNK^WT^ mice and modestly upregulated in JNK^KO^ mice (Cluster 2) or were not upregulated in JNK^KO^ mice (Cluster 3). Both clusters were enriched for metabolic pathways (*p*_adj_ < 10^−71^), whereas Cluster 2 showed additional enrichment for genes involved in RNA metabolism (Fig. 5d). There was no striking enrichment of pathways in Cluster 4, which contained genes downregulated in both JNK^WT^ and JNK^KO^ mice during involution (Fig. 5d). Thus, while JNK is dispensable for the regulation of a limited number of genes during involution (Cluster 4), the loss of JNK greatly affects the expression of other involution-associated genes (Clusters 1, 2, and 3).

The requirement of JNK for normal involution-associated gene expression (Fig. 5) may be a secondary consequence of delayed involution and suppression of this developmental program of gene expression. Alternatively, these involution-associated genes may be directly targeted by JNK. To explore these two possible mechanisms, we compared the presence of AP1 binding sites (defined by ENCODE ChIPseq analysis of cJUN and JUND [37]) near genes that are developmentally regulated during involution in a JNK-dependent manner with genes that are not significantly regulated by JNK. This analysis demonstrated significant enrichment of cJUN and JUND binding sites (*p* = 2 × 10^−16^) with ~35% of the JNK-regulated genes during involution (Figure S7). However, the remaining 65% of the JNK-regulated genes lack cJUN/JUND binding sites (Figure S7). The JNK-regulated expression of these genes may reflect targeting of other transcription factors by JNK or represent a consequence of delayed involution.

### JNK promotes the expression of genes that remodel the mammary gland during involution

It is established that matrix metalloproteinases (MMPs) are involved in remodeling the extracellular matrix [38]. Interestingly, differentially expressed genes with enrichment of cJUN/JUND binding include several *Mmp* genes that may contribute to mammary gland involution (Fig. 5c; Figure S7). Indeed, *Mmp3* is implicated in both mammary gland development and involution [38, 39]. We found that JNK deficiency markedly suppressed the increased *Mmp3* expression detected in JNK^WT^ mice on involution day 3 (Figure S8), consistent with the observation that JNK^KO^ mice exhibit delayed involution (Fig. 1; Figure S2). We also found that the expression of *Mmp2*, *Mmp3*, *Mmp9*, *Mmp11*, *Mmp12*, *Mmp13*, *Mmp14*, and *Mmp15* were significantly decreased in JNK^KO^ mice compared with JNK^WT^ mice after 3 days of involution (Figure S8).

Alterations in the tissue inhibitors of metalloproteases (TIMPs) could also impact mammary gland involution. Indeed, *Timp1* overexpression results in more rapid adipocyte repopulation of the gland, whereas *Timp3* loss can promote epithelial cell apoptotic signaling [40, 41]. Unexpectedly, we found that *Timp2* and *Timp3* expression was increased during involution in JNK^WT^ mice and that this was suppressed in JNK^KO^ mice (Figure S8).

Epithelial cell death during involution is caused, in part, by a lysosomal pathway mediated by Cathepsins [13], and it is established that increased *Ctsb* (encodes Cathepsin B) and *Ctsl* (encodes Cathepsin L) expression, together with reduced expression of *Serpina3g* (encodes the protease inhibitor Spi2A), contribute to STAT3-induced involution [9, 13]. Interestingly, the *Ctsb* gene binds the AP1 transcription factors cJUN/JUND and therefore might exhibit JNK-dependent expression (Fig. 5; Figure S7). Indeed, we found that the expression of *Ctsb* was increased on involution day 3 in JNK^WT^ mice and that this increased expression was suppressed in JNK^KO^ mice (Figure S9a). A similar pattern of expression was observed for *Ctsl* (Figure S9b). In contrast, JNK deficiency caused no significant change in *Serpina3g* expression during involution (Figure S9c). Thus, reduced Cathepsin expression may contribute to the delayed involution phenotype caused by JNK deficiency.

**Fig. 6:**
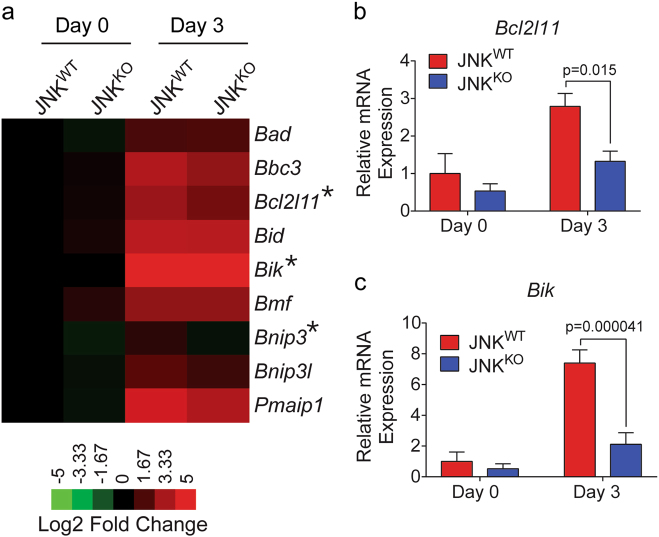
JNK deficiency suppresses the expression of pro-apoptotic BH3-only genes. **a** Heatmap representation of RNA-seq data showing pro-apoptotic BH3-only gene expression. Asterisks denote genes that are differentially expressed (*Bcl2l11* *q* = 0.021, *Bik* *q* = 0.04, and *Bnip3* *q* = 0.00015; calculated by applying the Benjamini–Hochberg method to the *p*-value) between JNK^WT^ and JNK^KO^ mammary glands on involution day 3 (mean; *n* = 3 JNK^WT^ mice and *n* = 3 JNK^KO^ mice for each condition). **b**, **c** Quantitative RT-PCR was performed on RNA isolated from mammary glands on involution day 0 and day 3. The relative expression of *Bcl2l11* (**b**), and *Bik* (**c**) mRNA was measured using Taqman® assays. Significance was calculated using two-way ANOVA with Bonferroni’s multiple comparisons test (mean ± SEM; day 0, *n* = 6 JNK^WT^ mice and *n* = 6 JNK^KO^ mice; day 3, *n* = 8 JNK^WT^ mice and *n* = 6 JNK^KO^ mice). *Bcl2l11* and *Bik* mRNA were significantly differentially expressed in both RNA-seq and quantitative RT-PCR assays

Epithelial cell death during involution is also caused by the mitochondrion-mediated apoptotic pathway induced by members of the BCL2 family [11, 14, 15]. Previous studies have implicated a key role for the BH3-only gene *Bcl2l11* (encodes BIM) in the promotion of epithelial cell death during the involution response [11, 15]. Moreover, it is established that the *Bcl2l11* gene is regulated by AP1 transcription factor binding to the promoter [42, 43]. We therefore anticipated that *Bcl2l11* gene expression may depend on JNK. Indeed, we found that involution caused increased expression of *Bcl2l11* mRNA in the mammary glands of JNK^WT^ mice during involution and that this response was suppressed in JNK^KO^ mice (Fig. 6a, b). The increased expression of *Bik* detected in JNK^WT^ mice was also suppressed in JNK^KO^ mice (Fig. 6a, c). In contrast, expression of the pro-apoptotic BH3-only genes *Bad*, *Bbc3, Bid*, *Bmf*, *Bnip3*, *Bnip3l*, and *Pmaip1* was similar in the involuting mammary glands of JNK^KO^ and JNK^WT^ mice (Fig. 6a; Figure S10).

Collectively, these data demonstrate that the delayed involution defect in JNK-deficient mice is associated with dysregulation of the gene expression program that promotes involution.

## Discussion

Weaning initiates the process of mammary gland involution that causes collapse of the epithelial cell compartment and its replacement by adipose tissue. Here we demonstrate that the JNK signaling pathway has a key role in the involution response. This program of mammary gland remodeling involves differential expression of 10,385 genes (Fig. 5). We show that 26% of this gene expression program requires JNK (Fig. 5).

Previous studies have demonstrated that involution is associated with activation of JNK [28] and increased AP1 transcription factor activity [28, 36]. Our analysis shows that JNK deficiency in the mammary epithelium suppresses the involution response mediated by increased expression of *Atf3*, *Fosl2*, *Jun*, and *Jund* (Fig. 4). Indeed, ~35% of JNK-regulated gene expression during involution is associated with the presence of AP1 binding sites (Figure S7). These data confirm that the JNK/AP1 signaling axis has an important role during involution [28, 36]. However, the remaining 65% of JNK-regulated expression during involution is not associated with genes in close proximity to AP1 binding sites. Some of this JNK-mediated regulation may be caused by AP1 binding to sites localized to distant enhancer elements, but there may also be roles for other JNK-regulated transcription factors [23]. It is also possible that some JNK-dependent gene expression may represent a consequence of a delayed involution response.

Examples of genes that may be directly targeted by JNK signaling during involution include matrix metalloproteases (Figure S8) that are regulated by AP1, including JUN [44, 45], and are implicated in both epithelial cell death [46] and adipocyte repopulation [40] during involution. Indeed, JNK-regulated *Mmp* expression may contribute to epithelial cell detachment during involution. A second example is represented by *Ctsb* and *Ctsl*, which encode Cathepsins that promote lysosomal cell death during involution and are targeted by AP1 (Fig. 5; Figure S7). A third example is represented by pro-apoptotic BH3-only members of the BCL2 family (Fig. 6) that can promote epithelial cell apoptosis during involution, including *Bcl2l11* that is required for normal involution [11, 15] and is a JNK/AP1 target gene [26, 42, 43]. Defects in the expression of these genes most likely contribute to the delayed involution observed in mice with JNK deficiency in the mammary epithelium (Fig. 1).

It is established that STAT3 is a key driver of the involution response [12]. Interestingly, STAT3 target genes that are required for cell death during involution, including *Ctsb* and *Bcl2l11*, are also targets of AP1 transcription factors. For example, the *Bcl2l11* promoter binds both STAT3 [11] and AP1 [26, 42, 43] at independent sites. The combinatorial actions of these transcription factors on the same promoter may lead to a synergistic increase in gene expression. This mechanism would account for the non-redundant functions of STAT3 and JNK/AP1 in the expression of these involution-related genes.

The discovery that JNK has a major role in mammary gland involution suggests that other members of the MAP kinase group of signaling proteins may also contribute to involution. Indeed, the ERK pathway is activated during early involution and may contribute to mammary gland remodeling [7]. For example, STAT3, a master regulator of involution, is phosphorylated and inhibited by ERK [47]. Moreover, the BH3-only protein BIM is required for involution [15] and is downregulated by ERK-mediated phosphorylation and ubiquitin-mediated degradation [32, 48]. Studies to test whether the ERK pathway contributes to involution are therefore warranted.

The p38 MAP kinases represent another group of MAP kinases that is implicated in involution [24]. It is established that p38α MAP kinase has a key role in luminal mammary epithelial cell fate by regulating RUNX1 expression in progenitor cells [49]. The p38 MAP kinase pathway therefore has an important role in mammary gland development. Moreover, p38 MAP kinase promotes epithelial cell anoikis and clearance of occluded mammary gland ducts by increasing the expression of the BH3-only protein BIM [25]. As BIM is required for normal mammary gland remodeling during involution [15], it is therefore possible that p38 MAP kinase contributes to the involution response. This prediction remains to be tested.

It is interesting that there are functional similarities between the JNK and p38 MAP kinases in mammary epithelial cells. For example, both p38 MAP kinase [25] and JNK [26] can promote mammary epithelial cell anoikis by increasing the expression of BIM by an AP1 transcription factor-dependent mechanism. It is likely that the non-redundant functions of p38 MAP kinase and JNK are mediated by different repertoires of AP1-related transcription factors. For example, ATF2 is preferentially phosphorylated and activated by p38 MAP kinase while JUN is phosphorylated by JNK in mammary epithelial cells [24]. Moreover, JNK is required for the expression of the AP1-related transcription factors ATF3, FOSL2, JUN, and JUND during involution (Fig. 4). It is likely that p38 MAP kinase leads to the activation of a different group of AP1-related transcription factors. JNK and p38 MAP kinase may therefore act in a non-redundant manner to regulate AP1-dependent gene expression. These separate signaling functions of p38 MAP kinase and JNK can lead to different pathological consequences; for example, p38 MAP kinase increases [49] and JNK decreases [27] mammary tumor development.

In summary, we show that JNK promotes mammary gland involution by a mechanism that is independent of changes in STAT3/5 or SMAD2/3 phosphorylation. Loss of JNK signaling causes delayed involution, reduced expression of AP1 transcription factors, and dysregulation of gene expression. Collectively, our analysis identifies JNK as a key signaling pathway that promotes mammary gland involution.

## Materials and methods

### Mice

We have previously described *Mapk8*^*LoxP/LoxP*^ mice and *Mapk9*^*LoxP/LoxP*^ mice [50, 51]. B6.129(Cg)-*Gt(ROSA)26Sor*^*tm4(ACTB-tdTomato,-EGFP)Luo*^/J mice [52] (RRID:IMSR_JAX:007676) and B6.Cg-Tg(Wap-cre)11738Mam/JKnwJ mice [35] (RRID:IMSR_JAX:008735) were purchased from Jackson Laboratories. Female mice were bred at age 10–12 weeks. Mammary glands from single parous females were harvested at 0, 3, 7, and 14 days after forced weaning following 9 days of lactation. The mice were housed in a specific pathogen-free facility accredited by the American Association of Laboratory Animal Care (AALAC). The animal studies were approved by the Institutional Animal Care and Use Committee at the University of Massachusetts Medical School.

### Genomic DNA analysis

The polymerase chain reaction (PCR) amplimers 5′-CTCTGCTGCCTCCTGGCTTCT-3′, 5′-CGAGGCGGATCACAAGCAATA-3′ and 5′-TCAATGGGCGGGGGTCGTT-3′ were used to detect presence of the *mTmG* (250 bp) and WT alleles (330 bp). The amplimers 5′-TTACTGACCGTACACCAAATTTGCCTGC-3′ and 5′-CCTGGCAGCGATCGCTATTTTCCATGAGTG-3′ were used to detect the *Cre*^+^ allele (450 bp). The amplimers 5′-AGGATTTATGCCCTCTGCTTGTC-3′ and 5′-GACCACTGTTCCAATTTCCATCC-3′ were used to detect the *Mapk8*^+^ (540 bp) and *Mapk8*^*LoxP*^ (330 bp) alleles. The amplimers 5′-GTTTTGTAAAGGGAGCCGAC-3′ and 5′-CCTGACTACTGAGCCTGGTTTCTC-3′ were used to detect the *Mapk9*^+^ (224 bp) and *Mapk9*^*LoxP*^ alleles (264 bp). The amplimers 5′-CCTCAGGAAGAAAGGGCTTATTTC-3′ and 5′-GAACCACTGTTCCAATTTCCATCC-3′ were used to detect the *Mapk8*^+^ (1550 bp), *Mapk8*^*LoxP*^ (1095 bp), and the *Mapk8*^∆^ alleles (395 bp). The amplimers 5′-GGAATGTTTGGTCCTTTAG-3′, 5′-GCTATTCAGAGTTAAGTG-3′, and 5′-TTCATTCTAAGCTCAGACTC-3′ were used to detect the *Mapk9*^*LoxP*^ (560 bp) and *Mapk9*^∆^ alleles (400 bp).

### Mammary gland analysis

Female mice were euthanized and mammary glands #2–5 were harvested, fixed in 10% formalin, dehydrated, and embedded in paraffin. Five micron-thick sections were cut and stained with hematoxylin and eosin (H&E) for analysis. Sections of #4 glands were also stained with antibodies against cleaved caspase-3 (1:100; Cell Signaling Technology Cat# 9662 RRID:AB_331439), phospho-STAT3 (1:400; Cell Signaling Technology Cat# 9145 RRID:AB_2491009), phospho-STAT5 (1:50; Abcam Cat# ab32364 RRID:AB_778105), phospho-SMAD2/3 (1:200; Santa Curz Biotechnology Cat# sc-11769-R), α-smooth muscle actin (Millipore Sigma Cat# A2547, RRID:AB_476701; 1:100 dilution), keratin 5 (BioLegend Cat# 905501 RRID:AB_2565050; 1:50 dilution), keratin 8 (DSHB Cat# TROMA-I RRID:AB_531826; 1:100 dilution), and GFP (Thermo Fisher Cat# A21311 RRID:AB_221477). Immunohistochemistry was performed using a biotinylated goat anti-IgG antibody (Biogenex Cat# HK340-5K) plus streptavidin-conjugated horseradish peroxidase (Vector Laboratories Cat# PK-6100) and 3,3’-diaminobenzidene (Vector Laboratories Cat# SK-4100). Sections were counter-stained with hematoxylin (Thermo Fisher). Images were acquired using a Zeiss Axiovert microscope. Immunofluorescence was performed using AlexaFluor 546 conjugated-goat anti-rabbit IgG (H + L) antibody (Thermo Fisher Cat# A11035 RRID:AB_143051), AlexaFluor 488 conjugated-goat anti-rabbit IgG (H + L) antibody (Thermo Fisher Cat# A-11008 RRID:AB_143165), AlexaFluor 633 conjugated-goat anti-mouse IgG (H + L) antibody (Thermo Fisher Cat# A-21052, RRID:AB_141459), AlexaFluor 633 conjugated-goat anti-rat igG (H + L) antibody (Thermo Fisher Cat# A-21094, RRID:AB_141553), or AlexaFluor 488 conjugated-goat anti-rat IgG (H + L) antibody (Thermo Fisher Cat# A11006 RRID:AB_141373), and counter-stained with 2-(4-amidinophenyl)-1H-indole-6-carboxamidine (DAPI). TUNEL staining was performed following the manufacturer’s recommendations (Sigma Cat# 11684795910). Fluorescence images were acquired using a Leica SP2 confocal microscope. Immunofluorescence staining of phospho-STAT3 was quantitated using ImageJ [53] on 10–15 images per mouse; the amount of phospho-STAT3 was normalized to keratin 8 or DAPI fluorescence and the mean value per mouse was calculated. Sections were examined in a blinded fashion for days 0, 3, 7, and 14 of involution. However, the marked histological differences on involution day 3 prevented blinded analyses.

### Immunoblot analysis

Tissue lysates from #2–3 glands were prepared using Triton lysis buffer (20 mM Tris (pH 7.4), 1% Triton X-100, 10% glycerol, 137 mM NaCl, 2 mM EDTA, 25 mM β-glycerophosphate, 1 mM sodium orthovanadate, 1 mM phenylmethylsulfonyl fluoride, and 10 μg/ml of aprotinin plus leupeptin). Extracts (30 µg) were subjected to immunoblot analysis with antibodies to STAT3 (Cell Signaling Technology Cat# 9139 RRID:AB_331757; dilution 1:1000), phospho-STAT3 (Cell Signaling Technology, Cat# 9145 RRID:AB_2491009; dilution 1:2000), and αTubulin (Sigma-Aldrich Cat# T5168; RRID:AB_477579). IRDye 680LT conjugated-donkey anti-mouse IgG antibody (LI-COR Biosciences Cat# 926-68022 RRID:AB_10715072) and IRDye 800CW conjugated-goat anti-rabbit IgG (LI-COR Biosciences Cat# 926-32211 RRID:AB_621843) were used to detect immune complexes, and these were quantitated using the Odyssey infrared imaging system (LI-COR Biosciences).

### Mammary epithelial cell isolation

Mammary epithelial cells were isolated as previously described [54, 55] with minor modifications [27]. Briefly, lymph nodes were removed and whole mammary glands were placed in DMEM/F12 supplemented with penicillin/streptomycin and nystatin. The glands were washed once in PBS before being minced and placed in DMEM/F12 containing 0.2% trypsin, 0.2% collagenase A, 5% fetal calf serum, and 5 µg/ml gentamicin (2 h) on a rotator at 37 °C. Cells and organoids were pelleted by centrifugation at 1500 rpm (10 min). The fatty layer was transferred to a second tube and dispersed with pipetting while the pellet was resuspended in DMEM/F12. The pellet and fatty layer were centrifuged again at 1500 rpm (10 min) and combined in one tube prior to incubation (2–5 mins at 25 °C with shaking) in DMEM/F12 supplemented with 10 µg/ml DNAse I. The cells were centrifuged at 1500 rpm (10 min) and resuspended in 10 ml of DMEM/F12. The epithelial cells and organoids were briefly (0.2 min) centrifuged at 1500 rpm 6–7 times and resuspended in fresh DMEM/F12 to wash out fibroblasts.

### RT-PCR analysis

The expression of *Bad* (Mm00432042_m1), *Bbc3* (Mm00519268_m1), *Bcl2l11* (Mm00437797_m1), *Bmf* (Mm00506773_m1), *Bid* (Mm00432073_m1), *Bik* (Mm00476123_m1), *Bnip3* (Mm01275601_g1), *Bnip3l* (Mm00786306_s1), and *Pmaip1* (Mm00451763_m1) mRNA and 18S RNA (4308329) was measured using TaqMan® assays using QuantStudio 12K Flex machine (Thermo Fisher). The amount of mRNA was normalized to the amount of 18S detected in the same sample.

### RNA-seq analysis

Mammary glands #2–5 were flash frozen in liquid nitrogen and RNA was isolated using the RNeasy kit with DNase treatment (Qiagen). RNA quality (RIN > 8) was confirmed using a Bioanalyzer 2100 (Agilent Technologies). Libraries were constructed according to the manufacturer’s instructions using the NeoPrep kit (Illumina). Paired-end RNA sequencing with reads (40 bp) were performed using a NextSeq500 (Illumina). Three independent libraries were analyzed for each condition. FastQC (version 0.10.1) [56] was used to generate sequence quality reports. Poor quality reads, adapter sequence and reads <20 bp were removed using Trimmomatic (version 0.36) [57]. The pre-processed Illumina paired-end Fastq datasets were aligned to the mouse reference genome (Ensembl GRCm38). Alignment was performed using Bowtie2 (v 2–2.1.0) [58] and Tophat2 (v 2.0.14) [59]. Samtools (version 0.0.19) [60] and IGV (version 2.3.60) [61] were used for indexing the alignment files and viewing the aligned reads respectively. Gene expression was quantitated as fragments per kilobase of exon model per million mapped fragments (FPKM) using Cufflinks (v 2.2.1) [62, 63] and differential expression was identified using the Cuffmerge and Cuffdiff tools. The false discovery rate (*q* value) was obtained by applying the Benjamini–Hochberg method to a *p*-value calculated using a one-tailed *t*-test [62, 63]. The library normalization method used for Cuffdiff was set to “classic-fpkm” and the dispersion method was set to “per-condition”. Cummerbund (version 2.4.1) [62] was used to assess replicate concordance between sample groups. Gene-set enrichment analysis was performed using differentially expressed gene lists with the WEB-based GEne SeT AnaLysis Toolkit (Webgestalt) [64] by selecting the KEGG database and viewing the 10 pathways with lowest *p*_adj_-value (*p*-value adjusted using the Benjamini–Hochberg method).

### Clustering analysis

The complex heatmap package (version 1.12.0) [65] was used to cluster RNA-seq data (Fig. 5c). The “clustering distance rows” parameter was set to “maximum”; the “clustering method rows parameter” was set to “ward.D”. Genes were included in the clustering analysis if they were differentially expressed (|log_2_ Fold Change| > 1; *q* < 0.01) between one or more pairwise group comparisons. Over 12,000 genes together with the gene expression levels were examined by *k*-means (*k* = 4) clustering.

### Enrichment analysis

We used RNA-seq data (Fig. 5) and Mouse ENCODE ChIPSeq datasets [37] (accession numbers GSM912901 and GSM912902) to evaluate the overlap between genes that were non-differentially expressed or differentially expressed between JNK^WT^ and JNK^KO^ on involution day 3 (Fig. 5c), and genes that bind the transcription factors cJUN and JUND. Peaks that passed the irreproducible discovery rate at a threshold of 2% were selected from the ENCODE project. The Mouse ENCODE ChIPSeq bed files with mm10 coordinates were entered as input into PAVIS [66] and the nearest genes to the peaks from the ChIPSeq data were annotated. The “genome assembly” and the “gene set” were set to “Ensembl GRCm38” and “mm10 all genes”, respectively. The upstream distance from the transcription start site and the downstream distance from the transcript termination site were each set to 20,000 bp. The intersections between the differentially expressed genes (identified by RNA-seq analysis (Fig. 5c)) and the genes that bind the transcription factors JUN and JUND (identified using ENCODE ChIPseq data) were identified using Interactivenn (http://www.interactivenn.net/) [67]. Statistical significance between two groups was determined by Pearson’s *χ*^2^ test.

### Statistical analysis

Data are presented as the mean and standard error. Statistical analysis was performed using GraphPad Prism version 7 (GraphPad Software, La Jolla). ANOVA with Bonferroni’s test was used to determine significance with an assumed confidence interval of 95%. The significance of pairwise comparisons was determined using Students *t*-test (*p* < 0.05). The false discovery rate (*q* value) was obtained by applying the Benjamini–Hochberg method to the *p*-value.

### Accession number

The RNA-seq data was deposited in the Gene Expression Omnibus (GEO) database with accession number GSE89495.

## Electronic supplementary material

Supplemental Material
