# Supplementary material for: The cJUN NH2-terminal kinase (JNK) pathway contributes to mouse mammary gland remodeling during involution
Source: Cell Death Differ. 2018 Mar 6;25(9):1702–15. doi: 10.1038/s41418-018-0081-z (PMC6143629; doi:10.1038/s41418-018-0081-z)
Supplement: Supplementary file 1 — Supplemental Material [file 41418_2018_81_MOESM1_ESM.pdf]

# Supplementary Information

## The cJUN $NH_2$-terminal kinase (JNK) pathway contributes to mouse mammary gland remodeling during involution

Nomeda Girnius, Yvonne J. K. Edwards, and Roger J. Davis

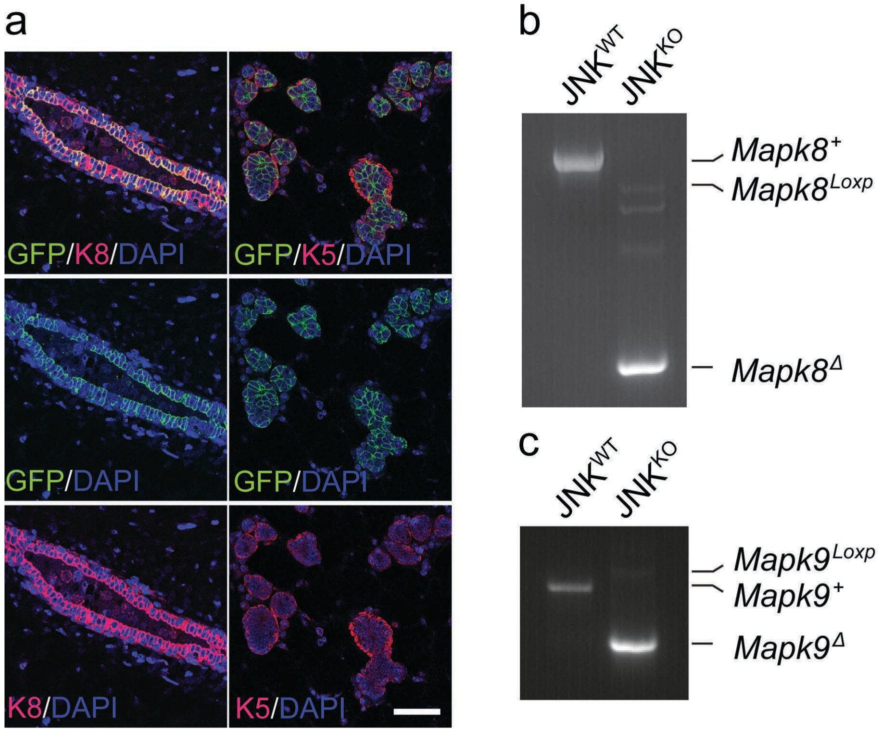


**Supplemental Figure S1. *Mapk8* and *Mapk9* gene disruption in luminal mammary epithelial cells.**

(**a**) Sections of mammary glands prepared from single parous female *Wap-Cre*$^{-/+}$ *Rosa*$^{mT/mG}$ reporter mice were stained with antibodies to GFP, keratin 5 (K5), or keratin 8 (K8), and then counter-stained with DAPI. The GFP antibody stains *Cre*$^{+}$ cells in the reporter mice. The images presented are representative of sections prepared from mammary glands of 7 mice. Scale bar = 50 µm.

(**b**,**c**) *Cre*-mediated recombination of *Mapk8*$^{LoxP}$ (b) and *Mapk9*$^{LoxP}$ (c) alleles was confirmed by PCR analysis of genomic DNA isolated from mammary epithelial cells isolated from mice after 10 days of lactation.

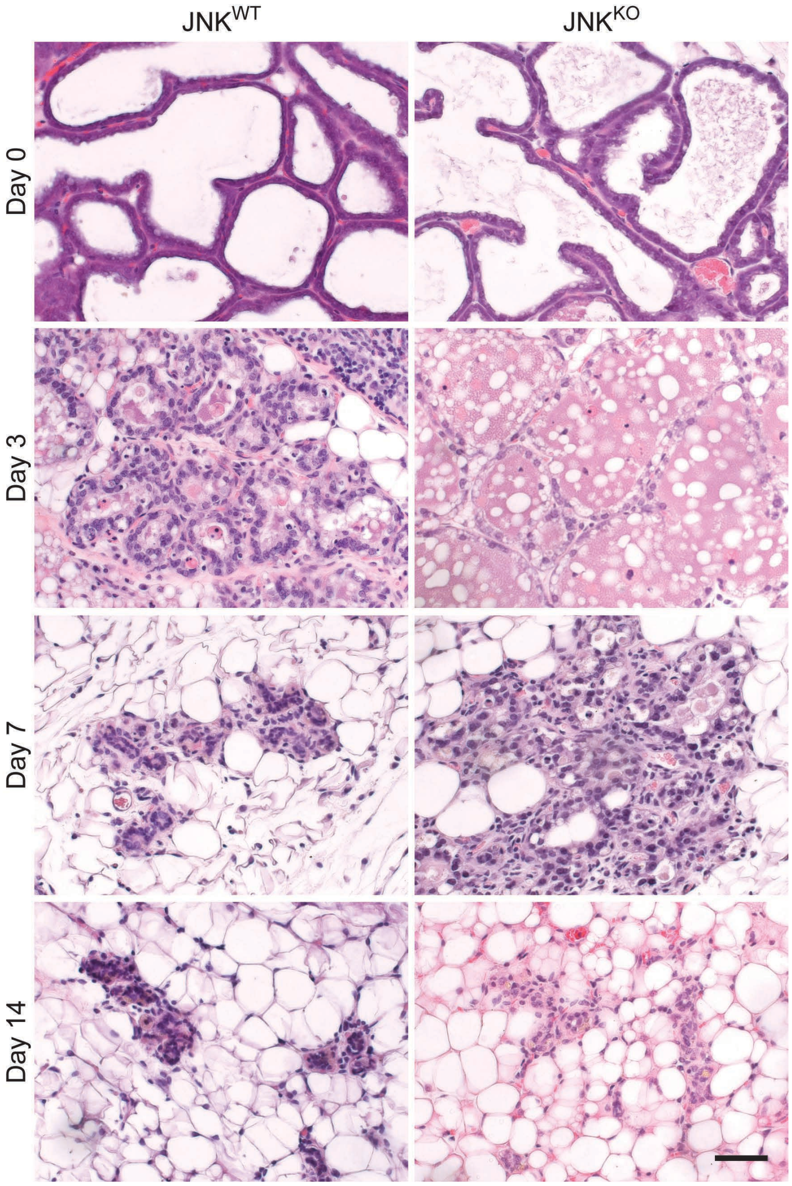


**Supplemental Figure S2. JNK is required for mammary gland involution.**

Sections of mammary glands from JNK$^{WT}$ and JNK$^{KO}$ female mice on day 0, 3, 7, and 14 of involution were stained with H&E. The images are representative of sections taken from n=5 JNK$^{WT}$ mice and n=5 JNK$^{KO}$ mice for each condition. Scale bar = 50 μm.

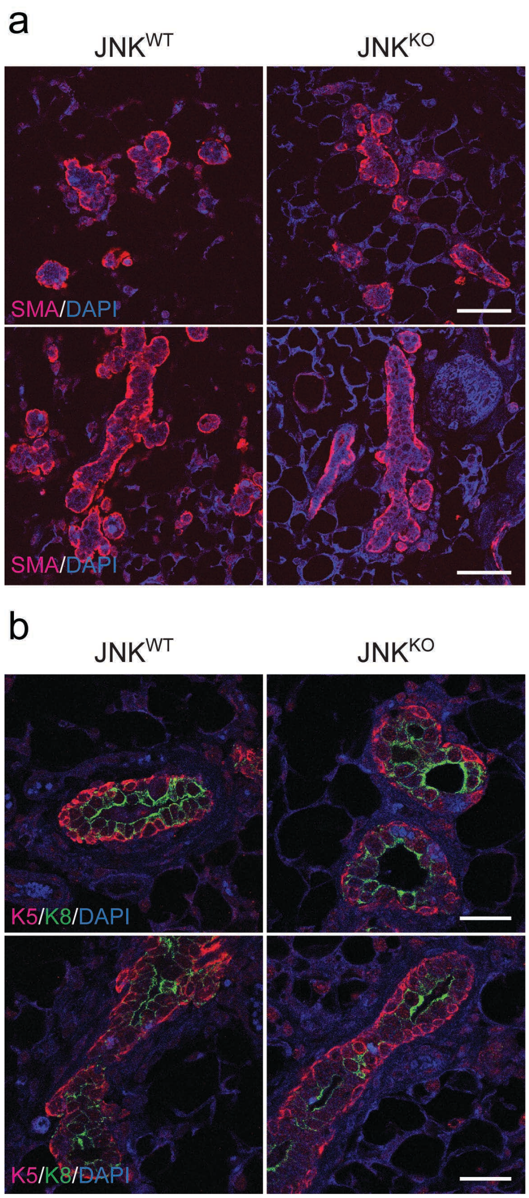


**Supplemental Figure S3. Epithelial cell populations in mammary glands after involution.**

(**a,b**) Mammary gland sections were stained with antibodies to smooth muscle actin (SMA, a) or with antibodies to keratin 5 and 8 (K5 and K8, b), and counterstained with DAPI. Representative images are presented (n=4 JNK$^{WT}$ mice and n=4 JNK$^{KO}$ mice). Scale bars = 50 μm (a) and 30 μm (b).

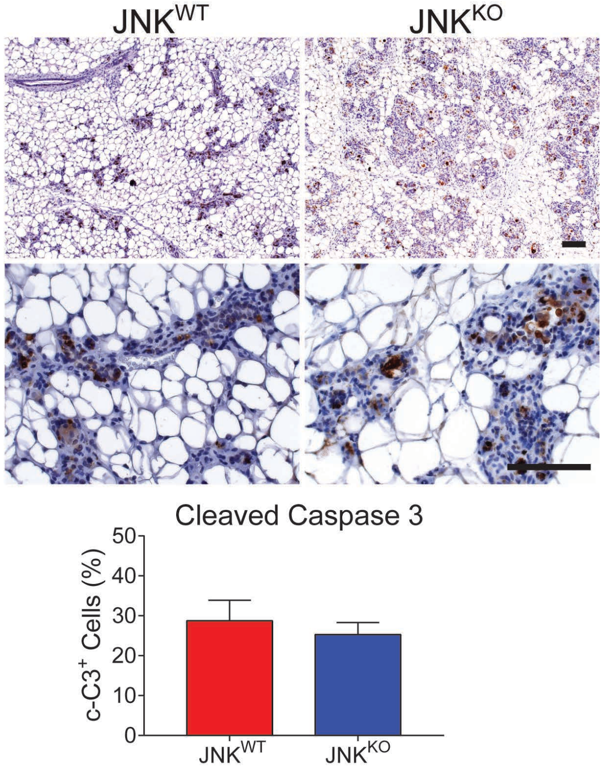


**Supplemental Figure S4. Caspase 3 activation in mammary glands after 7 days of involution.**

Sections prepared from mammary glands of $JNK^{WT}$ mice and $JNK^{KO}$ mice on day 7 of involution were stained with an antibody to cleaved caspase 3 ($c\text{-}C3^{+}$). Representative images are presented. $c\text{-}C3^{+}$ cells were quantitated in 6 fields (40x) per section and presented as the % of total cells. Statistical significance was calculated using an unpaired, two-tailed t-test (mean ± SEM; n=5 $JNK^{WT}$ mice and n=5 $JNK^{KO}$ mice; p=0.59). Scale bars = 100 μm.

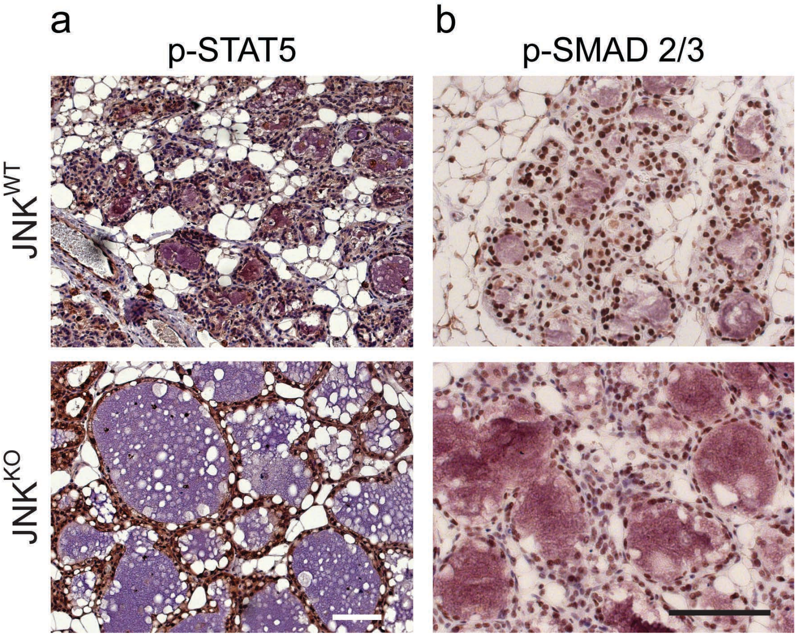


**Supplemental Figure S5. JNK deficiency does not alter STAT5 or SMAD2/3 activation during involution.**

(**a**,**b**) Sections prepared from mammary glands of 5 $JNK^{WT}$ mice and 5 $JNK^{KO}$ mice on involution day 3 were stained with an antibody to p-STAT5 (a) or p-SMAD2/3 (B) and counterstained with hematoxylin. Representative images are presented. Scale bars = 100 μm.

a

| Biological Group | | Mean Reads | Mean Read Mapping Rate |
|---|---|---|---|
| $JNK^{WT}$ | Day 0 | 66,439,000 | 98.3 % |
| $JNK^{KO}$ | Day 0 | 63,476,000 | 98.1 % |
| $JNK^{WT}$ | Day 3 | 61,129,000 | 97.9 % |
| $JNK^{KO}$ | Day 3 | 64,328,000 | 98.0 % |

b

$JNK^{WT}$

| Biotype | Frequency | Differentially Expressed |
|---|---|---|
| Protein Coding | 22,580 | 9,882 |
| Pseudogene | 5,850 | 413 |
| lincRNA | 1,762 | 108 |
| Processed Transcript | 621 | 57 |
| Antisense | 1,443 | 32 |
| miRNA | 1,768 | 11 |
| snoRNA | 1,453 | 10 |
| misc RNA | 529 | 4 |
| Polymorphic pseudogene | 15 | 3 |
| rRNA | 299 | 2 |
| Mt tRNA | 8 | 1 |
| Sense Intronic | 86 | 1 |
| Sense Overlapping | 8 | 1 |
| | 36,422 | 10,525 |

c

$JNK^{KO}$

| Biotype | Frequency | Differentially Expressed |
|---|---|---|
| Protein Coding | 22,583 | 9,468 |
| Pseudogene | 5,850 | 457 |
| lincRNA | 1,762 | 94 |
| Processed Transcript | 621 | 51 |
| Antisense | 1,443 | 33 |
| snoRNA | 1,453 | 9 |
| miRNA | 1,768 | 8 |
| misc RNA | 529 | 6 |
| Polymorphic pseudogene | 15 | 4 |
| rRNA | 299 | 3 |
| Sense Intronic | 86 | 1 |
| Sense Overlapping | 8 | 1 |
| | 36,417 | 10,135 |

**Supplemental Figure S6. Summary of RNA sequencing analysis.**

(**a**) RNA sequencing analysis of mammary glands isolated from $JNK^{WT}$ and $JNK^{KO}$ mice is summarized. Each biological group comprises 3 independent replicates.

(**b,c**) Biotypes were determined for both detected gene expression and genes that are differentially expressed ($log_2$|Fold Change|>1, q<0.01) between day 0 and day 3 of involution in $JNK^{WT}$ mice (b) and $JNK^{KO}$ mice (c).

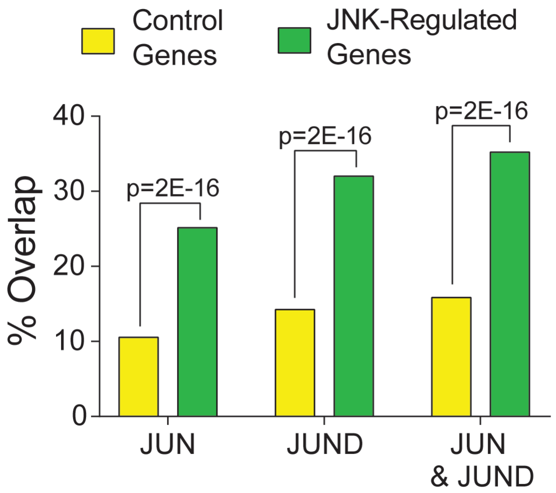


**Supplemental Figure S7. Enrichment of AP1 binding sites with genes that exhibit JNK-dependent expression during involution.**

Genes expressed in a JNK-dependent manner (green) or JNK-independent manner (yellow) after 3 days of involution (Figure 5) were compared with genes identified by ChIP-Seq analysis (cJUN, JUND, or JUN plus JUND) to determine overlap between JNK-dependent gene expression and genes with AP1 binding sites. Pearson's Chi-squared test was used to determine statistical significance.

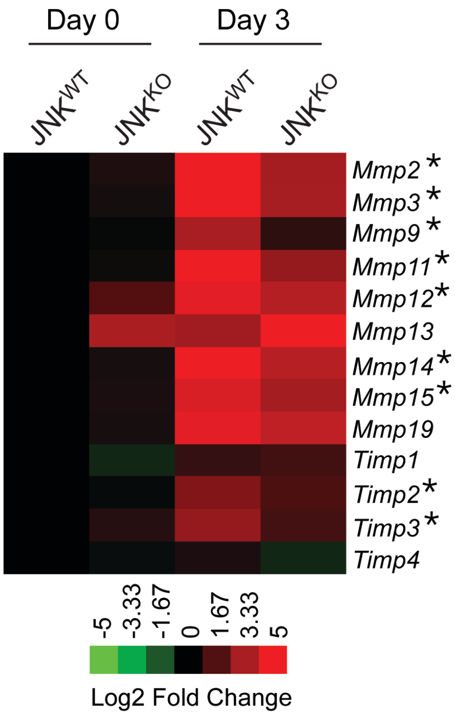


**Supplemental Figure S8. Mammary gland expression of *Mmp* and *Timp* genes.**

Heatmap representation of RNA-seq data showing *matrix metalloproteinase* and *tissue inhibitor of metalloproteinase* gene expression. Asterisks denote genes that are differentially expressed (*Mmp2* q=0.00015, *Mmp3* q=0.00015, *Mmp9* q=0.00015, *Mmp11* q=0.00015, *Mmp12* q=0.0041, *Mmp14* q=0.00015, *Mmp15* q=0.00029, *Timp2* q=0.027, *Timp3* q=0.00042; calculated using the Benjamini-Hochberg method) between $JNK^{WT}$ and $JNK^{KO}$ mammary glands on involution day 3 (mean; n=3 $JNK^{WT}$ mice and n=3 $JNK^{KO}$ mice for each condition).

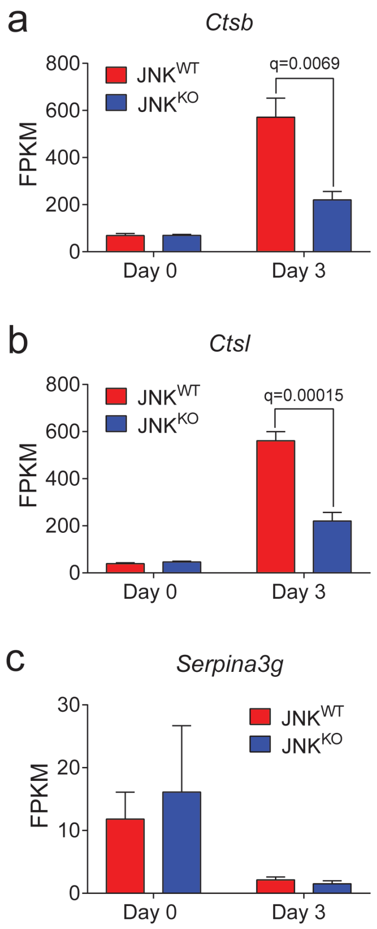


**Supplemental Figure S9. Mammary gland expression of cathepsin.**

(**a**-**c**) The mRNA expression of *Ctsb* (a), *Ctsl* (b), and *Serpina3g* (c) was measured by RNA-seq analysis. The data are presented as fragments per kilobase of exon model per million mapped fragments (FPKM) (mean ± SEM; n=3 $JNK^{WT}$ mice and n=3 $JNK^{KO}$ mice). The Benjamini-Hochberg method was used to calculate q-values.

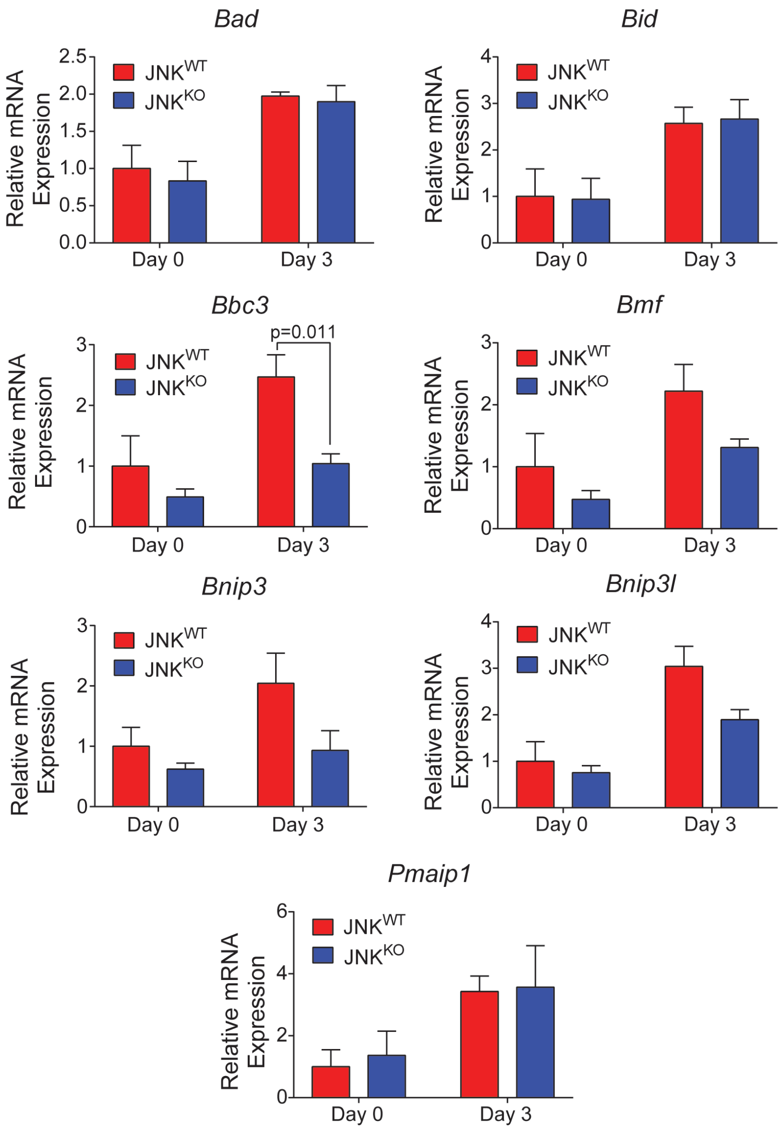


**Supplemental Figure S10. Mammary gland expression of BH3-only genes.**

Quantitative RT-PCR was performed on RNA isolated from mammary glands on involution day 0 and day 3. The relative expression of *Bad*, *Bbc3*, *Bid, Bmf, Bnip3, Bnip3l, and Pmaip* mRNA was measured using Taqman® assays. Statistical significance was calculated using two-way ANOVA with Bonferroni's multiple comparisons test (mean ± SEM; Day 0, n=6 $JNK^{WT}$ mice and n=6 $JNK^{KO}$ mice; Day 3, n=8 $JNK^{WT}$ mice and n=6 $JNK^{KO}$ mice; p-values <0.05 are presented).
